# Factors Contributing to Sex Differences in Mice Inhaling *Aspergillus fumigatus*

**DOI:** 10.3390/ijerph17238851

**Published:** 2020-11-28

**Authors:** Andrea L. Schaefer, Mai Ceesay, Jennicca A. Leier, Jacob Tesch, Brian D. Wisenden, Sumali Pandey

**Affiliations:** Biosciences Department, Minnesota State University Moorhead, Moorhead, 56563 MN, USA; schaefer2017@gmail.com (A.L.S.); ceesayma@mnstate.edu (M.C.); jennicca.leier@go.mnstate.edu (J.A.L.); Jacob.tesch.2020@gmail.com (J.T.); wisenden@mnstate.edu (B.D.W.)

**Keywords:** airway remodeling, collagen, mucus, principal component analysis, IgE

## Abstract

*Aspergillus fumigatus* is a respiratory fungal pathogen and an allergen, commonly detected in flooded indoor environments and agricultural settings. Previous studies in Balb/c mice showed that repeated inhalation of live and dry *A. fumigatus* spores, without any adjuvant, elevated allergic immune response and airway remodeling. Sex-specific differences can influence host-pathogen interactions and allergic-asthma related outcomes. However, the effect of host sex on immune response, in the context of *A. fumigatus* exposure, remains unknown. In this study, we quantified the multivariate and univariate immune response of C57BL/6J mice to live, dry airborne *A. fumigatus* spores. Our results corroborate previous results in Balb/c mice that repeated inhalation of live *A. fumigatus* spores is sufficient to induce mucus production and inflammation by day 3 post last challenge, and antibody titers and collagen production by day 28 post-challenge. Principal Component Analysis (PCA) showed that females exhibited significantly higher levels of immune components than males did. Taken together, our data indicate that host-sex is an important factor in shaping the immune response against *A. fumigatus*, and must be considered when modeling disease in animals, in designing diagnostics and therapeutics for *A. fumigatus*-associated diseases or while drafting evidence-based guidelines for safe mold levels.

## 1. Introduction

While several federal agencies guide the public on health effects associated with environmental fungal exposure and on ways to mitigate it, there are no federally accepted health-based standards for safe fungal levels as reported by the United States Government Accountability Office (U.S. GAO) [1]. Diseases like cancer and farmer’s lung, have implicated mold-associated exposures [2,3,4]. *Aspergillus fumigatus* is one such environmental fungus, which is efficiently cleared from the lungs of an immunocompetent host, but can cause a variety of diseases in others, such as Severe Asthma with Fungal Sensitization (SAFS), Allergic Bronchopulmonary Aspergillosis (ABPA), Chronic Necrotizing Pulmonary Aspergillosis (CNPA), and Invasive Aspergillosis (IA). The severity of infection/colonization can range from mortality associated with invasive aspergillosis, and ongoing morbidity associated with allergic conditions [5]. 

Exposure to this saprophytic fungus is ubiquitous in the environment, as it is commonly found in soil and air, and plays an important role in carbon and nitrogen recycling. Exposure to *A. fumigatus* is particularly concerning in intensive care units [6,7], flooded indoor environments [8,9,10], and agricultural surroundings [11,12,13]. *A. fumigatus* is commonly detected in grain dust and agricultural environments, and antibodies against its allergens have been detected in farmers [11,13,14]. As a result, repeated inhalation of high quantities of mold and their antigens over an extended period is likely [15,16]. Besides humans, *A. fumigatus* is a major respiratory pathogen in birds, and infection by *A. fumigatus* may induce significant economic losses particularly in turkey production [17]. Due to the aforementioned reasons, *A. fumigatus* remains a significant public health burden and the problem is further complicated due to emerging azole resistance [18,19,20]. 

The airborne nature of fungal spores makes the exposure unavoidable and ubiquitous in most circumstances. Therefore, animal models that mimic repeated, nose-only human exposure to dry fungal spores are critical in informing exposure-related guidelines and policies, and the design of therapeutics and diagnostics for the clinical settings. Due to a significant public health burden associated with *A. fumigatus,* animal models are critical in establishing the underlying cellular and molecular mechanisms. We and others have previously shown that repeated pulmonary exposure to live *A. fumigatus* spores without an adjuvant can increase airway remodeling, including mucus production, collagen deposition and epithelial cell hypertrophy [21,22,23]. These studies in mouse models were significant as they showed that repeated exposure to *A. fumigatus* spores in a manner that mimics the nose-only human exposure can induce allergic asthma-related histopathological changes. The outcome of *A. fumigatus* interaction with a host is shaped by several elements of the immune response, including neutrophils, antigen presenting dendritic cells, opsonizing antibodies, and CD4^+^T cells [24]. In an allergic host, IgE antibodies and eosinophils perpetuate allergic inflammation and airway remodeling [25,26], and serum IgE titers serve as diagnostic aids in allergic bronchopulmonary aspergillosis [27]. 

While epidemiological studies have implicated differences in sexes with regards to allergic asthma and infections, mouse-based studies have underreported sex-associated differences, in the context of *A. fumigatus* exposure [28,29]. The effect of host-sex on immune-related cells, proteins and histopathological changes have not been investigated in the context of *A. fumigatus* exposure. Biological sex affects innate and adaptive immune responses, resulting in sex differences in autoimmunity, allergic asthma and response to infections and vaccines. These sex-specific differences stem from several superimposing elements, including genomic and epigenomic organization, as well as a direct effect of sex steroid (estrogen, progesterone and testosterone) on components of the immune system [30,31,32,33,34,35,36]. 

In this study, we investigated the immune response of C57BL/6J mice to live, airborne *A. fumigatus* spores, and used sex and timepoint (days 3 and 28 post third fungal challenge) as predictor variables. Since the immune response to *A. fumigatus* is complex and dynamic, and is shaped by multiple host-related parameters, we used multivariate (principal component analysis (PCA)) and univariate analysis to quantify the immune response. 

## 2. Materials and Methods 

### 2.1. Mice

Male and female C57BL/6J mice were purchased from The Jackson Laboratory (Sacramento, CA, USA) and housed in the colony in Langseth Hall at Minnesota State University Moorhead (Moorhead, MN, USA). The mice were housed with sex- and age-matched mates, in microfilter-topped cages (Ancare, Bellmore, NY, USA), under constant room temperature, in a 12 h light/dark cycle, and were fed ad-libitum. All inhalational exposures were initiated in mice at 6 weeks of age. All protocols were in accordance with MSUM’s Institutional Animal Care and Use Committee (IACUC protocol#19-R/T-BIO-030-N-N-B) guidelines.

### 2.2. Aspergillus fumigatus Inhalation in Mice

**Figure 1:**
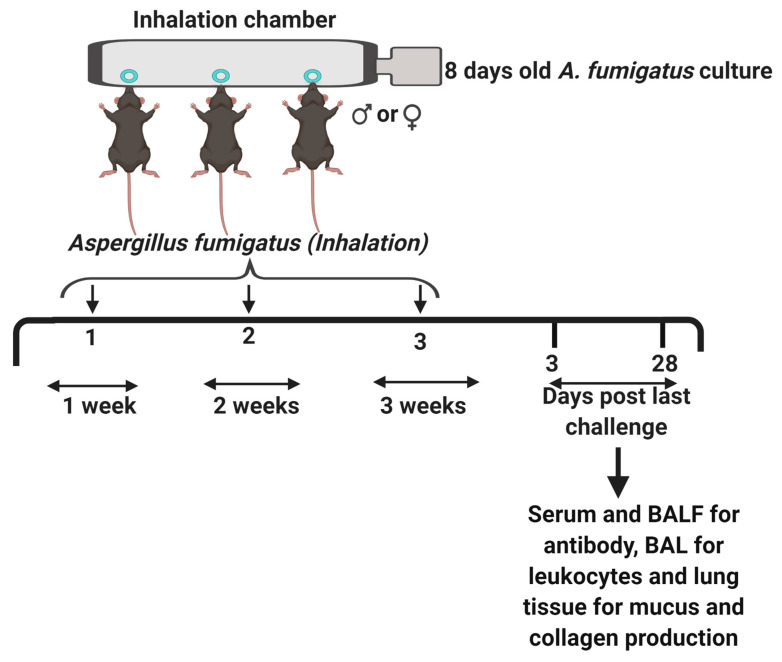
The study design. Six weeks old male or female C57BL/6J mice were anesthetized with an intraperitoneal injection of ketamine and xylazine, and laid in a supine position with their nostrils sticking into the ports of the inhalation chamber [11]. An 8-day-old culture of *Aspergillus fumigatus* on Saboraud Dextrose Agar was hooked to the air supply on the right end of the chamber and steady airflow blew off the dry, live *A. fumigatus* spores, which were inhaled by the mice. This challenge was repeated for 10 min., once per week for three weeks. Naïve (untreated) mice were maintained as controls. The mice were euthanized at days three or twenty-eight post third fungal challenge. Serum and bronchoalveolar lavage fluid (BALF) were collected and stored at -20 °C for antibody analysis. The bronchoalveolar lavage (BAL) was cytospun and stained with Quick-Dip stains for leukocyte analysis and whole left lungs were fixed, sectioned at 5 µm and stained with Sirius Red/Fast Green or Periodic Acid Schiff stains for collagen or mucus/goblet cell metaplasia, respectively. The figure was created with BioRender.com.

*Aspergillus fumigatus* (strain NIH 5233) was obtained from American Type Culture Collection, lyophilized, reconstituted and stored at 4 °C, as per manufacturer’s instructors. An aliquot of *A. fumigatus* spores was spread onto Saboraud Dextrose Agar media. The *A. fumigatus* culture was allowed to mature for 8 days at 37 °C, before being used for murine challenge. Male and female mice without any prior exposure to fungal antigens (naïve mice), were challenged with dry, live and airborne *Aspergillus fumigatus* spores, using a previously described spore delivery method (Figure 1) [21,37]. Briefly, mice were anesthetized with an intraperitoneal injection of ketamine (75 mg/kg) and xylazine (25 mg/kg) before a 10 min, nose-only inhalation of live *A. fumigatus* conidia. After three weekly exposures with *A. fumigatus*, mice were euthanized with an overdose of sodium pentobarbitone on days 3 or 28 post third fungal challenge. Naïve (unexposed) mice were euthanized at day 0 and maintained as controls throughout the study. All fungal work was carried by trained personnel wearing task appropriate Personal Protective Equipment (N95 masks, safety goggles, a lab coat and gloves) in Class II biological safety cabinets, and was followed by thorough disinfection protocols using Spore-Klenz (Steris Life Sciences, OH, USA) and 70% *v/v* ethanol. 

### 2.3. Bronchoalveolar Lavage Cell Counts

The trachea was cannulated and 1 mL sterile PBS was used to lavage the bronchoalveolar space of the mouse. The lung wash was centrifuged at 2000× *g* at 4 °C for 10 min and the supernatant was stored at −20 °C for protein analysis (hereafter called bronchoalveolar lavage fluid (BALF)). The cell pellet was resuspended in 200 µL of sterile PBS and cytospun using Cytospin 4 (ThermoFisher Scientific, Waltham, MA, USA). The cytospun cells were stained using quick-dip stain (ThermoFisher Scientific, Waltham, MA, USA), as per manufacturer’s protocol. Two trained lab members identified and counted leukocytes from these slides, from five random fields at 1000X magnification, in a blinded manner.

### 2.4. Antibody Analysis

Antibody levels were analyzed in serum and BALF. The BALF was obtained as described in the previous section. For serum, approximately 0.3 mL of mouse blood was centrifuged at 13,000× *g* at 4 °C, and the supernatant was stored at −20 °C until further analysis. IgE and IgG_2a_ levels were analyzed in 100 fold diluted serum or undiluted BALF, using mouse isotype-specific ELISA kits (BD Biosciences, Inc., San Jose, CA, USA), as per manufacturer’s directions. 

### 2.5. Histological Analysis

Whole left lungs were fixed in Carnoy’s solution (Ward’s Science, St. Catharines, ON, Canada) for 4 h, followed by 70% ethanol overnight. The lungs were paraffin-embedded, sectioned at 5 µm thickness, and stained with Sirius Red/Fast Green or Periodic Acid Schiff stains for collagen or mucus/goblet cell metaplasia, respectively. Collagen was identified as pinkish-red thread-like structures around the airways on Sirius Red/Fast Green stained slides. Mucus/goblet cells were identified within the terminal airways, as pinkish-purple stains against a greenish-blue background on Periodic Acid Schiff stained slides. The slides were scanned using the Motic Digital Slide Scanner (Richmond, British Columbia, Canada), and images were captured and analyzed using the Motic Digital Slide Assistant System Lite 1.0. with 20X magnification (original magnification = 200X, scale bar = 60 µm) software. Five terminal airways per mouse section were analyzed and scored on a three-point scale for collagen or mucus production, by two blinded personnel, with 0 = no collagen/mucus, 1 = low collagen/mucus, 2 = medium collagen/mucus and 3 = high collagen/mucus. 

### 2.6. Statistical Analysis

Because immune responses to infection involve multiple coordinated components, measuring immune system responses as a multivariate response is appropriate to quantify an integrated response. We applied principal components analysis (PCA) to capture and distill the majority of the information contained in nine components of the immune response (mucus production/goblet cells, IgG_2a_ serum, IgG_2a_ BALF, IgE serum, collagen deposition, and absolute counts of macrophages, lymphocytes, neutrophils, eosinophils) into two, new variables called principal component axis 1 (PC1) and principal component axis 2 (PC2) using SPSS v.26 (International Business Machines Corporation, Armonk, NY, USA). This analysis was followed up by univariate comparisons between sexes and timepoints using non-parametric Mann–Whitney tests or Kruskal–Wallis tests, respectively, using GraphPad Prism 8 (GraphPad Software, Inc., San Diego, CA, USA). All statistical tests were two-tailed with alpha set at 0.05.

## 3. Results

### 3.1. PCA: Multivariate Analysis of Immune Response to A. fumigatus

**Figure 2:**
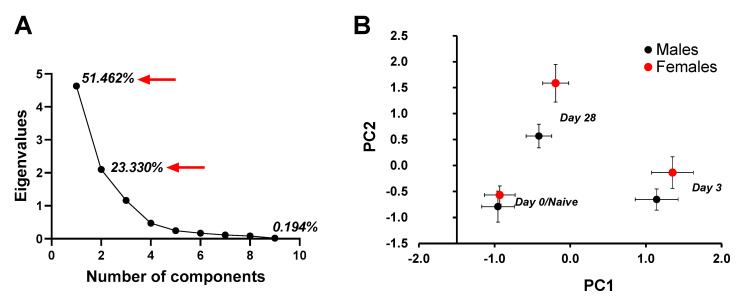
Principal Component Analysis to assess the effect of different factors on immune response mounted by male and female mice at days 0 (naïve), 3 or 28 post third *A. fumigatus* challenge. (**A**) Eigenvalues for 10 principal components showed that the first two components had eigenvalues greater than 1 and captured 74.792% of the variance. (**B**) Principal Component 1 versus 2 graph showed different clusters for day 0 (naïve), 3 and 28 mice. Male and female mice clustered differently on all timepoints (*p* value = 0.004), and the distance between the two sexes increased with time post-challenge (*p* < 0.001).

The first and second principal components together captured 74.8% of the variation contained within the nine original variables (Figure 2A). PC1 (51.462% variation explained) correlated positively with neutrophils, eosinophils, lymphocytes and mucus production, and negatively with macrophages (Table 1). PC2 (23.33% variation explained) positively correlated with collagen, IgE serum, IgG_2a_ serum, and IgG_2a_ BALF. Using PC1 and PC2 as response variables, we tested for an effect of sex and timepoint. There was a significant effect of timepoint (day 0, 3 or 28 post third fungal challenge) on PC1 (*F*_2, 36_ = 123.265, *p* < 0.001), but no effect of mouse’s sex (*F*_1, 36_ = 1.674, *p* = 0.204) and the interaction between sex and timepoint was not significant (*F*_2, 36_ = 0.297, *p* = 0.745). For PC2, there was a significant effect of timepoint (*F*_2, 36_ = 39.417, *p* < 0.001), and a significant sex effect (*F*_1, 36_ = 11.456, *p* = 0.002). The interaction between sex and timepoint was not significant (*F*_2, 36_ = 1.795, *p* = 0.181). A plot of PC1 versus PC2 (Figure 2B) showed clear spatial separation of data clusters for males and females and day (Day0/Naïve, Day 3 and Day 28), where females ranked higher on PC2 loaded components on each of the listed days. Using PC1 as a covariate in an ANCOVA, PC2 showed a significant effect of sex (*F*_1, 35_ = 9.579, *p* = 0.004) and timepoint (*F*_2, 35_ = 39.942, *p* < 0.001), while the interaction between sex and timepoint was not significant (*F*_2, 35_ = 1.586, *p* = 0.219). Taken together, a multivariate approach to quantify a coordinated multicomponent immune response indicated an effect of timepoint (Day 0/Naïve, Day 3, Day 28) on variables that load significantly onto PC1 (neutrophils, eosinophils, mucus, lymphocytes and macrophages) and PC2 (collagen, IgE serum, IgG_2a_BALF and IgG_2a_serum). An effect of sex was observed only on the variables that load significantly onto PC2, showing that immune response by females was more intense than the response by males in terms of collagen, IgE serum and IgG_2a_ serum and IgG_2a_ BALF.

### 3.2. Univariate Analyses of Individual Components of Immune Responses to Repeated Inhalation of A. fumigatus

#### 3.2.1. Antibody Titers 

**Figure 3:**
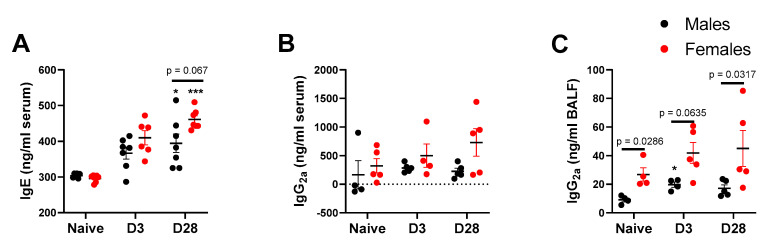
Antibody titers (mean ± SEM) in serum (**A** and **B**) or bronchoalveolar lavage fluid (**C**) obtained from male and female mice at days 0 (naïve), 3 or 28 post third *A. fumigatus* challenge. *, ***; *p*-value ≤ 0.05 and 0.001, respectively, indicate a comparison of mean ± SEM values between day 0 (naïve) and *A. fumigatus* challenged mice. The solid line and corresponding *p*-values indicate the pairwise comparisons between the sexes at each timepoint.

With repeated inhalation of *A. fumigatus,* both male and female mice showed a progressive increase in IgE titers over days 3 and 28 post-third fungal challenge (Figure 3A), indicating allergic sensitization via nose-only exposure. More importantly, female mice showed a higher (*p* = 0.067), albeit not statistically significant, IgE titer than male mice and naïve mice at day 28 post-last fungal challenge (Figure 3A). While noticeable differences in IgE titers were observable in serum, we did not notice a significant change in Bronchoalveolar Lavage Fluid (BALF) titers for IgE, relative to naïve mice (data not shown). While IgE antibody is considered as a typical hallmark of allergy, IgG_2a_ titers usually correspond with Th-1 type immune response. In this study, the serum IgG_2a_ levels trended towards higher titers in female mice challenged with *A. fumigatus*, but the differences failed to reach statistical significance (Figure 3B). As compared to male mice, BALF IgG_2a_ titers (Figure 3C) were significantly higher in naïve and *A. fumigatus* challenged female mice. For the three antibodies, the levels start increasing in response to *A. fumigatus* exposure, at day 3 post last fungal challenge, and significant increases are observable by day 28, indicating the perpetuation of adaptive immune response, which may continue beyond day 28. Throughout, female mice challenged with *A. fumigatus* showed higher antibody titers (Figure 3).

#### 3.2.2. Leukocyte Counts 

**Figure 4:**
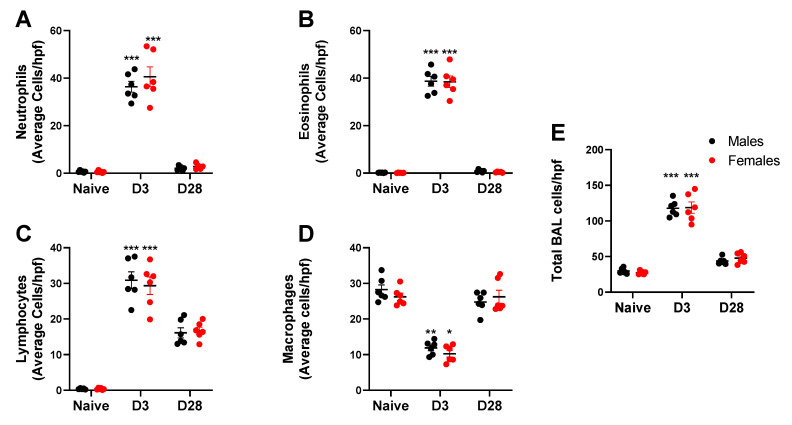
Leukocyte counts (mean ± SEM) observed in the bronchoalveolar lavage (BAL) wash obtained from male and female mice at days 0 (naïve), 3 or 28 post third *A. fumigatus* challenge. *, **, ***; *p*-value ≤ 0.05, 0.01 and 0.001, respectively, indicate a comparison of mean ± SEM values between day 0 (naïve) and *A. fumigatus* challenged mice. Average cells per high power field (hpf) are represented in (**A**) Neutrophils; (**B**) Eosinophils; (**C**) Lymphocytes; (**D**) Macrophages; (**E**) Total BAL cells

Repeated inhalation of *A. fumigatus* caused male and female mice to significantly increase neutrophil (Figure 4A), eosinophil (Figure 4B) and lymphocyte (Figure 4C) counts and decrease the macrophage counts on day 3 post-challenge (Figure 4D). By day 28, granulocytes were down to baseline, while lymphocytes and macrophages (Figure 4C,D) predominated the BAL cell fraction (Figure 4E). While the inflammation peaked at day 3 and dropped by day 28, there was no significant effect of mouse sex on the absolute BAL cell counts. While absolute counts are useful, fold change may provide insights into the biological significance of the data. We computed the fold change in leukocyte counts in *A. fumigatus* challenged male and female mice, with respect to naïve mice (Table 2). As compared to naïve mice, there was a 4-fold increase in total leukocyte count in *A. fumigatus* challenged male and female mice, at day 3 post-challenge. The baseline was restored (<2-fold change) by day 28 post-challenge. While there was no difference in total inflammation fold change between male and female mice, the neutrophils (70 versus 52-fold), eosinophils (462 versus 258-fold) and lymphocytes (80 versus 53-fold) fold changes were significantly higher in *A. fumigatus* challenged female mice, as compared to male mice, at day 3 post-challenge. The fold change in lymphocytes (46 versus 28-fold), but not eosinophils, neutrophils or macrophages, was significantly higher in *A. fumigatus* challenged female mice, as compared to male mice, at day 28 post-challenge. Interestingly, the drop in macrophage with *A. fumigatus* inhalation at day 3 post challenge was minimal (<1-fold change) and was not significantly different between male and female mice (Table 2). 

#### 3.2.3. Airway Remodeling 

**Figure 5:**
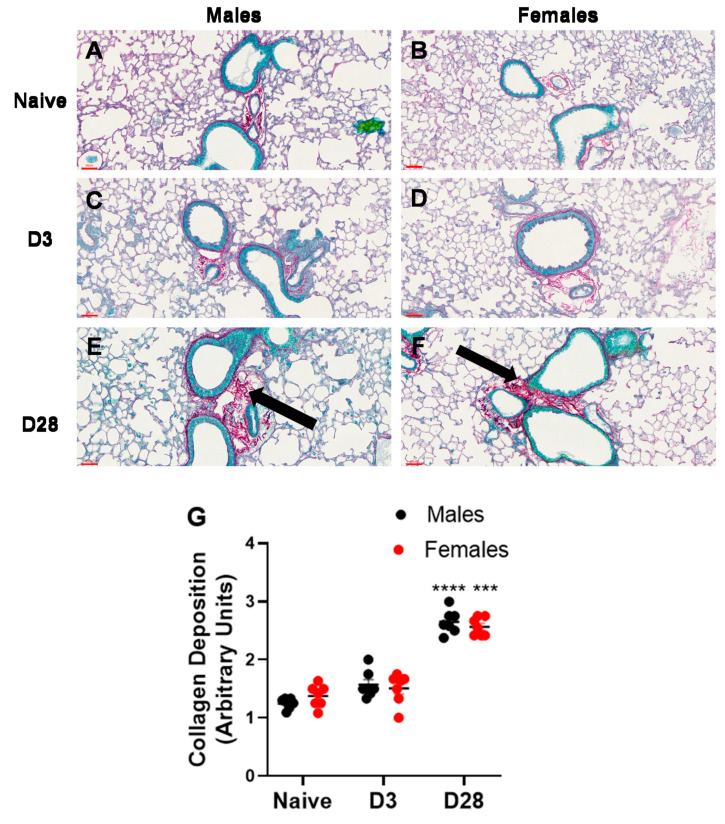
Collagen production observed in lung sections obtained from male and female mice at days 0 (naïve), 3 or 28 post third *A. fumigatus* challenge. The collagen deposition (pinkish red thread like structures indicated by black arrows) around five random terminal airways were scored for each mouse, by two blinded personnel, and the mean ± SEM values are reported in (**G**). The scale bars in (**A**–**F**) represented by red lines correspond to 60 µm. ***, ****; *p*-value ≤ 0.001 and 0.0001, respectively, indicate the comparison of day 0 (naïve) and *A. fumigatus* challenged mice.

**Figure 6:**
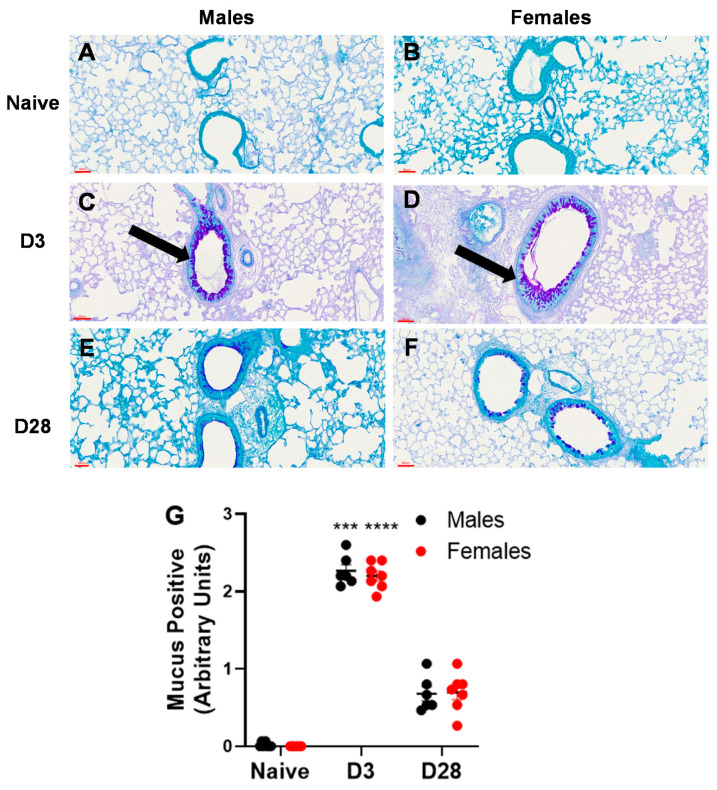
Mucus production observed in lung sections obtained from male and female mice at days 0 (naïve), 3 or 28 post third *A. fumigatus* challenge. The mucus production and goblet cell metaplasia (pinkish-purple stain indicated by black arrows) around five random terminal airways were scored for each mouse, by two blinded personnel, and the mean ± SEM values are reported in (**G**). The scale bars in (**A**–**F**) represented by red lines correspond to 60 µm. ***, ****; *p*-value ≤ 0.001 and 0.0001, respectively, indicate the comparison of day 0 (naïve) and *A. fumigatus* challenged mice.

Airway remodeling is a structural change in the lung, which can lead to progressive loss of lung function. We measured two markers of airway remodeling, namely, collagen deposition and mucus production/goblet cell metaplasia. With repeated inhalation of *A. fumigatus,* both male and female mice showed a significant increase in collagen deposition (Figure 5) and mucus production (Figure 6), as compared to naïve mice. While minimal collagen was observed in naïve (Figure 5A,B) and *A. fumigatus* challenged male (Figure 5C) and female (Figure 5D) mice at day 3 post last challenge, the collagen deposition peaked at day 28 (Figure 5E,F) for mice of both sexes (graphical representation in Figure 5G). Similarly, mucus production was not observed in male (Figure 6A) and female (Figure 6B) naïve mice and it peaked at day 3 post *A. fumigatus* challenge in male (Figure 6C) and female (Figure 6D) mice. While male and female mice were positive for mucus production at day 28 post *A. fumigatus* challenge (Figure 6E,F), the differences were not statistically significant as compared to the naïve mice (graphical representation in Figure 6G). With PCA, PC2 identified collagen as a measure that differed between the sexes (Figure 2B, Table 1), the univariate analysis did not pick any significant difference in sexes in terms of collagen or mucus production.

## 4. Discussion

Exposure to dry spores of *Aspergillus fumigatus* is ubiquitous in the environment, and particularly severe in farm environments or indoor settings post-flooding. Animal models that mimic human fungal exposure are indispensable in providing mechanistic insights into several features associated with *A. fumigatus* exposure, including humoral or cell-mediated immune response and airway remodeling. These animal models have helped to address the pathophysiological processes underlying allergy or infection, and provide a platform for assessing diagnostic and therapeutic effects of antifungal and anti-allergic drugs [28]. Thus, a thorough knowledge of factors that may affect the simulation of human exposure in a mouse model is critical to establish standardized protocols and generate usable, reproducible data.

Since the immune response to *A. fumigatus* is complex and dynamic, and can be shaped by several host-related parameters, we adopted a principal component analysis (PCA) approach to quantify the multivariate immune response and identify trends and patterns in the data. While PCA is commonly used in sequencing and ecological data analysis, this statistical analysis is a powerful tool for detecting coordinated multifaceted immune responses [38], and one that is more sensitive to detect multicomponent effects than conventional univariate tests applied to individual components of an immune response. For example, the effects of sex on collagen, IgG_2a_ BALF, and IgG_2a_ serum were not detectable by univariate Mann–Whitney tests but were readily identified as a significant component of a sex difference from the factor loadings on PC2. 

Among factors that may affect the outcome of an experimental study in mice, sex is important, yet an under-studied factor. In terms of *A. fumigatus* infection, a recent review reported that animal sex was not reported in 152 articles, and both sexes were used without differentiation in 32 studies [29]. Previous allergic asthma based models have investigated sex differences [39,40,41,42,43,44,45,46,47]; however, almost all of these studies investigated immune responses to either ovalbumin or house-dust mite allergen. *A. fumigatus* is unique because unlike ovalbumin, it is a clinically relevant allergen and a pathogen. Live *A. fumigatus* spores are capable of secreting proteases and can engage Pattern-Recognition Receptors via Pathogen Associated Molecular Patterns (PAMPs), and therefore, *A. fumigatus-*based models may not use an adjuvant to initiate an allergic response in mice [48]. The pathological changes (e.g., airway remodeling) in response to repeated inhalation of *A. fumigatus* spores are mediated by *A. fumigatus* (e.g. fungal viability) and host-related (e.g. allergic status) factors [21,49]. 

Sex-based differences have also been explored in epidemiological studies, which show that prevalence, severity and frequency of asthma are higher in adult females than in males [50,51]. In human females aged >18 years, the prevalence rate of asthma is 9.8% versus 5.5% in adult males. This is the opposite of the situation pre-puberty (age <18 years), where males are more frequently affected than females (8.3% in boys versus 6.7% in girls) [52]. In terms of *A. fumigatus* infection, Steeg et al.’s review anecdotally reported a male bias in prevalence, incidence and severity, with males being more susceptible to infection than females [35]. While epidemiological studies, focused on humans, provide evidence for sex-based differences, animal models are instrumental in delineating the underlying mechanisms and establishing the cause and effect relationships.

Serum IgE is a classical marker for allergic sensitization in diagnostic settings. Amongst the five isotypes, IgE is the least abundant isotype and has the lowest serum half-life of ~12 h [53]. The IgE effector functions are activated by its binding to Fc receptors FcɛRI and FcɛRII/CD23 [54]. Previous studies have shown that IgE antibodies triggered against *A. fumigatus* antigens can degranulate mast cells [55], and the biologics (Omalizumab) targeting IgE show variable effects in the clinic [56]. We observed serum IgE antibody titers to be elevated in female mice (*p* = 0.067) challenged with *A. fumigatus*, relative to naïve and male mice, at day 28 post-challenge. While our result did not reach statistical significance, the trend is very strong for females and correlates with previous studies in mouse models of ovalbumin or house dust mite based allergy [40,42]. While IgE production is associated with Th-2 type response and IL-4 production [57], IgG_2a_ production is typically associated with Th-1 type response [58,59]. Since *A. fumigatus* is known to induce Th-1 and Th-2 type response [22], and localized antibody repertoires are therapeutically significant [60,61], we measured IgG_2a_ in serum and BALF. A similar trend was observed for IgG_2a_ in serum and BALF, with females showing higher titers than males, in naïve and challenged mice. Sex-specific differences in humoral immunity have also been observed in human adults with females showing greater antibody responses than males, higher basal immunoglobulin levels, and higher B cell numbers [62,63,64]. Evolution-based hypothesis have been put forth to explain elevated humoral immunity in females. Fink and Klein postulated that natural selection favors increased antibody production in females compared with males because the transfer of maternal antibodies from mother to offspring through placenta and milk is critical for its survival and ensures reproductive success [65]. Kelly Lee and others argued that humoral (antibodies, B and Th-2 lymphocytes) immune functions are energetically and nutritionally less costly than cell-mediated and innate immune responses, since it does not involve systemic activation [66,67,68]. Therefore, generally, female mammals who invest energy in reproduction are likely to mount stronger antibody and weaker cell-mediated immune responses [68,69].

Airway remodeling is explained by several histopathological changes in the lung, including goblet cell metaplasia, subepithelial collagen deposition, epithelial cell hyperplasia and increased smooth muscle thickness. It accounts for the irreversible, persistent airflow obstruction in some asthmatic patients, and can be mediated by several inflammatory and epigenetic pathways [70]. Mouse based studies are critical to identifying these mechanisms and causative factors to inform the design of effective therapeutics. We have previously shown that airway remodeling associates with increased granulocytic inflammation caused by repeated inhalation of live *A. fumigatus* spores [21]. Inhalation of dead *A. fumigatus* spores was not as potent as live spores in eliciting collagen deposition but could increase mucus production in mice that were skewed towards allergic sensitization due to prior exposure to fungal antigens via subcutaneous/intraperitoneal route. In this study, we observed similar trends for mucus and collagen production, with mucus production peaking at day 3 and collagen production peaking at day 28 post-challenge. However, we did not detect an effect of sex on mucus or collagen deposition with the univariate analysis. However, PCA analysis showed that the mucus production and collagen deposition correlated strongly with PC1 and PC2, respectively. PC1 was significantly influenced by day (naïve/day 0, day 3 and 28 post challenge) but not sex. PC2 was significantly influenced by day and sex, and females showed an increased response on PC2 components, relative to the males, and this divergence between males and females increased in the following order: naïve < day 3 < day 28. 

Pulmonary infections with *A. fumigatus* have been shown to induce immune responses characterized by granulocytes [21] and Th1, Th2 and Th17 cells [22]. While both neutrophils and alveolar macrophages can mediate antifungal defense [71], early recruitment of neutrophils is critical to prevent an invasive fungal disease and host mortality [72,73]. Also, neutrophils are strongly associated with severe fungal asthma, which responds poorly to inhaled corticosteroids [74,75]. In addition to neutrophils, eosinophils’ role in antifungal defense has been investigated [76,77,78]. Eosinophil counts in the blood and bronchi increase in *Aspergillus fumigatus* associated asthma, and eosinophils secrete a variety of mediators to orchestrate airway remodeling in an allergic lung [26]. In the current study, both neutrophils and eosinophils increased drastically in response to *A. fumigatus*, and accordingly, we did not observe any hyphal filaments in murine lungs, with Periodic Acid Schiff stain. 

The fold change data for granulocyte counts indicated that the increase was significantly higher at early time point in females than in males. While the effect of sex steroids on neutrophils is less known, previous studies have shown higher eosinophil counts in females, as compared to males in OVA-sensitized mice [79], and the eosinophil recruitment was negatively correlated with estrogen receptor-β activation [80]. Our study also showed an increased fold change in lymphocytes in *A. fumigatus* challenged female mice than in male mice at early and late timepoints. While sex-specific differences in leukocyte counts were apparent in fold change data, the absolute counts did not differ significantly between males and females. We think that it is important to consider both fold change and absolute counts while drawing meaningful conclusions. For example, while absolute macrophage counts seem to be dropping at early timepoints with *A. fumigatus* challenge, the fold change was <1 fold, which was minimal as compared to the fold change in granulocytes and lymphocytes, which ranged between 52 and 462 fold at day 3-post challenge. Thus, future experiments with flow cytometry and functional assays should confirm the biological significance of sex-associated differences in leukocyte count.

The C57BL/6 mice and Balb/c are known to vary in T-helper type response, with C57BL/6 mice being Th-1/IFN-γ predominant and Balb/c mice being Th-2/IL-4 predominant [81]. Despite these differences, previous studies showed no significant differences in susceptibility to lethal systemic *A. fumigatus* infection [82]. Similarly, no strain-specific differences in clearance of a sub-lethal fungal dose from liver, kidneys and spleen were observed [83,84], and the lack of difference was attributed to the ability of both strains to increase IFN-γ and IL-17 levels in response to sub-lethal *A. fumigatus* infection [83]. However, strain-dependent differences were observed in liver injury and fungal clearance from the lungs, after a sublethal systemic *A. fumigatus* infection [84]. The strain specific differences in the context of pulmonary *A. fumigatus* infection are not well-established. While we compared the immune response of male and female C57BL/6J mice in this study, we have reported results from a study in Balb/cJ mice previously, albeit not in a sex-specific manner [21]. Our results corroborate previous results in Balb/c mice that repeated inhalation of live *A. fumigatus* spores is sufficient to induce mucus production and inflammation by day 3 post-challenge, and antibody titers and collagen production by day 28 post-challenge. In this study, female C57BL6/J mice exhibited significantly higher levels of immune components than males did. Future studies will focus on a side-by-side comparison between male and female Balb/c and C57BL/6 mice to establish the strain and sex-specific differences to pulmonary *A. fumigatus* infection. 

## 5. Conclusions

*A. fumigatus* associated diseases present a significant public health burden, and the airborne nature of fungal spores makes the exposure unavoidable and ubiquitous in most circumstances. In this study, we mimicked human nose-only exposure to dry, live fungal spores in mice and identified sex and time to be significant predictors of the immune response against live *A. fumigatus*. The multivariate analysis (Principal Component Analysis) showed that female mice exhibit a higher immune response than the males, and the divergence between the two sexes increased in the following order: naïve < day 3 < day 28. The univariate analysis showed female mice to be associated with higher antibody titers and fold change in granulocytes and lymphocytes. Therefore, this study provides evidence that sex and timepoint should be maintained as critical and separate variables when modeling the disease in animals, to establish standardized protocols and generate usable, reproducible data. Sex-related differences associated with *A. fumigatus* exposure will also critically inform the design of diagnostics and therapeutics for *A.fumigatus*-associated diseases or while drafting evidence-based guidelines for safe mold levels. Future investigations should dive into the underlying mechanisms (epigenetic and hormonal) for sex-associated differences in the context of *A. fumigatus* exposure. 

## Figures and Tables

**Table 1:** The factor matrix for principal component analysis.

Principal Components		
Item	1	2
Neutrophils	0.955	−0.189
Eosinophils	0.953	−0.237
Mucus	0.935	−0.057
Lymphocytes	0.906	0.127
IgE serum	0.437	0.764
IgG2a BALF	0.294	0.594
IgG2a serum	0.113	0.622
Collagen	−0.053	0.787
Macrophages	−0.908	0.212

Note: The column values representing the factor loadings (correlation coefficients) show that neutrophils, eosinophils, mucus and lymphocytes were strongly, positively correlated with PC1 and macrophages were negatively correlated with PC1. PC2 variance showed a strong positive correlation with IgE serum, collagen, IgG_2a_ serum and IgG_2a_ BALF.

**Table 2:** Average fold change observed in leukocyte count in the bronchoalveolar lavage wash obtained from male and female naïve or *A. fumigatus* challenged mice at days 3 or 28 post third fungal challenge.

Cell Type	Male Naïve	Female Naïve	Male D3	Female D3	Male D28	Female D28
Macrophages	1	1	−0.4	−0.4	0.9	1.0
Neutrophils	1	1	52.0	69.6 ^#^	2.9	6.9
Eosinophils	1	1	257.7	461.9 ^##^	5.6	9.4
Lymphocytes	1	1	53.0	80.1 ^#^	27.7	45.5 ^##^
Total	1	1	4.0	4.3	1.5	1.7

Note: The fold change was calculated relative to the naïve cell counts. ^#^, ^##^; *p*-value ≤ 0.05 and 0.01, respectively, indicate a comparison of average fold change values between male and female *A. fumigatus* challenged mice. The negative sign indicates a decrease in fold-change in *A. fumigatus* challenged mice with respect to naïve mice.
